# Inverse sex-based expression profiles of PTEN and Klotho in mice

**DOI:** 10.1038/s41598-020-77217-5

**Published:** 2020-11-19

**Authors:** Natalia Prudente de Mello, Diana Zukas Andreotti, Ana Maria Orellana, Cristoforo Scavone, Elisa Mitiko Kawamoto

**Affiliations:** 1 Laboratory of Molecular and Functional Neurobiology, Department of Pharmacology, Institute of Biomedical Sciences 1, University of São Paulo, Avenida Professor Lineu Prestes, 1524, São Paulo, 05508-000 Brazil; 2 Laboratory of Molecular Neuropharmacology, Department of Pharmacology, Institute of Biomedical Sciences, University of São Paulo, São Paulo, Brazil

**Keywords:** Cell biology, Neuroscience

## Abstract

Sex differences are considered predictive factors in the development of several neurological diseases, which are also known to coincide with impaired phosphoinositide 3-kinase (PI3K)-AKT pathway activity, an essential signaling cascade involved in the control of several cellular functions such as autophagy and apoptosis. Here, under physiological conditions, we show important sex differences in the underlying balancing mechanisms that lead to similar AKT activity levels and autophagy and apoptosis processes in the two sexes. We demonstrate inverse sex-based expression of PTEN and Klotho, two important proteins that are known to negatively regulate the AKT pathway, and inverse sex-dependent levels of mTOR and FoxO3a activity. Taken together, our findings indicate that inverse sex-based regulation may be one of the underlying balancing mechanisms that differ between the sexes and a possible cause of sex-based autophagic and apoptotic responses to triggering situations that can lead to a sex-based predisposition to some neurological diseases.

## Introduction

Over time, several studies have taken place to determine the underlying mechanisms related to the development of neurological diseases, especially to understand how the central nervous system (CNS) responds under pathological conditions. However, considering that many neurological diseases start long before the onset of the symptoms, understanding the physiological system when it is first altered may provide valuable information for further understanding the causes. More specifically, understanding sex-related differences, well-known risk factors for diseases such as autism spectrum disorder (ASD), Parkinson’s disease (PD) and Alzheimer’s disease (AD)^1–5^, can contribute to the identification of possible new targets for drug development.

During development, the phosphoinositide 3-kinase (PI3K)/AKT pathway has an essential role in cellular growth and proliferation, as well as in many other processes, such as autophagy and apoptosis, that maintain the physiological system^6,7^. Due to the importance of this pathway, alterations to key regulators of it, such as the phosphatase and tensin homolog deleted on chromosome 10 (PTEN), have been previously observed in ASD patients^8,9^, in addition to some other diseases in which sex is a predictive factor^10,11^. Similarly, Klotho, an anti-aging protein, also regulates the AKT pathway by inhibiting insulin signaling and consequently negatively regulating AKT activity^12^. Similar to that in PTEN, the reduction in Klotho expression has been linked to the development of some diseases, especially those that are age-related, such as AD^13,14^. In fact, these age-related pathologies are known to have impaired autophagy and apoptosis processes^15^, which are regulated, among others, by mTOR and FoxO3a, important downstream targets of the AKT pathway.

In the present study, we aimed to identify the physiological differences in the PI3K-AKT pathway of female and male mice that may contribute to the understanding of the underlying mechanisms related to the differences in neurological disease prevalence between sexes. Here, we show, for the first time, an inverse expression profile of PTEN and Klotho proteins in female and male mice. Furthermore, we demonstrate that AKT is not phosphorylated differently between the sexes, nor are autophagic and apoptotic proteins phosphorylated differently by sex, even though we observed that downstream targets such as mTOR and FoxO3a showed different activity levels depending on sex. Thus, our findings suggest that under physiological conditions, PTEN and Klotho may act together in a sex-dependent manner that maintain the similarities in the levels of AKT activity and important biological processes, such as autophagy and apoptosis, in both male and female physiological systems. Our results may contribute to understanding the sex-based differences in autophagy and apoptosis under altered conditions, such as AKT hyperactivation, which can further activate or inhibit basal mTOR and FoxO3a activity levels.

## Results

### Female mice express higher PTEN protein levels in the brain than males

**Figure 1:**
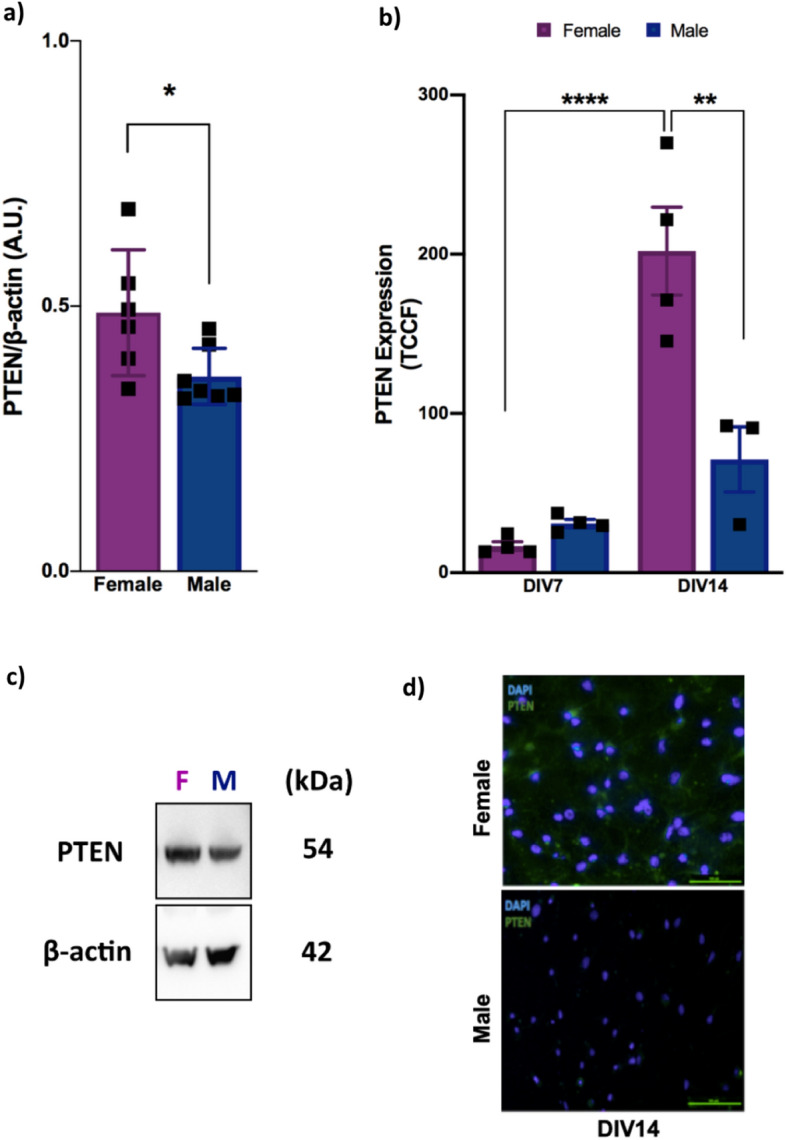
Female mice express higher levels of PTEN protein in the brain than males. (**a**) Cortical PTEN protein expression in 2-month-old female and male wild-type mice as analyzed by western blotting [n = 7 (male) and n = 6 (female); *P = 0.0335, ^#^q = 0.0410; two-tailed Student’s *t*-test], (**b**) embryonic cortical neurons from male and female wild-type embryos 7 days in vitro (7 DIV) and 14 days in vitro (14 DIV) as analyzed by immunofluorescence [(n = 4 (male and female); each time, 50 cells of each embryo were counted; ****P < 0.0001; **P = 0.0014); two-way ANOVA followed by Tukey’s post hoc test. (**c**) Representative image of PTEN and β-actin bands in male and female adult mice. (**d**) Representative images of PTEN staining of male and female mice 14 DIV (scale bar, 100 µm; 20× magnification). All results are expressed as the means ± S.E.M. *P < 0.05, **P < 0.01, ****P < 0.0001. Full-length blots are provided in Supplementary Fig. S1. Multiple *t* tests comparisons were corrected by controlling FDR. The summary with all statistical analyses are provided in Supplementary Material Table 1.

To investigate possible sex differences in the expression of key proteins in the CNS, PTEN cortical expression was analyzed in both male and female adult mice. PTEN was expressed in female mice at a significantly higher level than it was in males (*P* < 0.05) (Fig. 1a,c). Since PTEN is essential for brain development, we sought to determine whether this difference is observed at an earlier stage of the animal’s life by PTEN immunostaining of embryonic neuronal cortical cells from both sexes after 7 and 14 days in vitro (DIV). We found that at different times, the PTEN expression was increase from 7 to 14 DIV (F(1, 11) = 43.73, *P* < 0.0001 for the time point factor)*,* showing that time had a significant effect on PTEN expression. In addition, when considering sex, PTEN expression was significantly higher in the neurons from the female embryos than it was in neurons from males (F(1, 11) = 11.71, *P* = 0.0057 for sex) (Fig. 1b,d). Taken together, these findings show that female mice express higher PTEN levels than male mice, and this expression seems to develop over time since no differences were observed in earlier stages of life (F(1, 11) = 18.13, *P* = 0.0013 for interaction factor). These data strongly indicate a greater need for this protein in the female adult biological systems and supports the argument for sex-based regulation of PTEN expression, in addition to highlighting a sexual dimorphism already present in embryonic cells.

### Sex inversely influences mTOR and FoxO3a activity in an AKT-independent pathway

**Figure 2:**
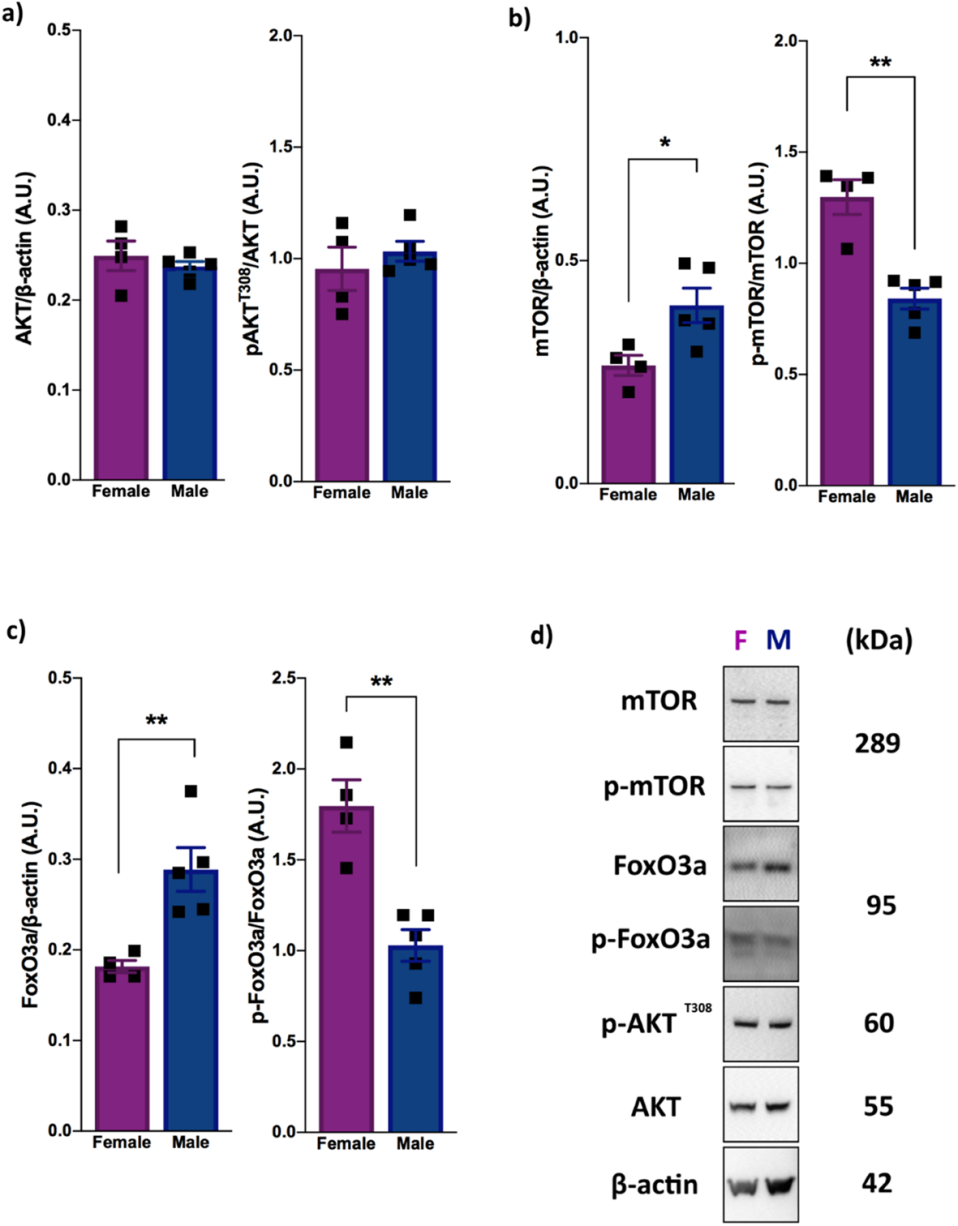
Female and male mice have inverse physiological mTOR and FoxO3a activity profiles in an AKT-independent pathway. (**a**) Phosphorylated AKT expression [n = 5 (male) and n = 4 (female); ns]. **(b**) Level of mTOR protein to β-actin [n = 5 (male) and n = 4 (female); *P* = 0.0261, ^#^q = 0.0384], and level of phosphorylated mTOR protein to total mTOR [n = 5 (male) and n = 4 (female); *P* = 0.0012, ^#^q = 0.0073]. (**c**) Level of FoxO3a protein to β-actin [n = 5 (male) and n = 4 (female); *P* = 0.0065, ^#^q = 0.0159], and level of phosphorylated FoxO3a protein to total FoxO3a [n = 5 (male) and n = 4 (female); *P* = 0.0020, ^#^q = 0.073] (**d**) Representative blot images showing p-mTOR, p-FoxO3a and p-AKT^T308^ expression in the male and female mice. All proteins were analyzed in 2-month-old female and male wild-type animals by western blotting. The data were analyzed by two-tailed Student’s *t*-test. All results are expressed as the means ± S.E.M. **P < 0.01. Full-length blots are provided in Supplementary Fig. S2. Multiple *t* tests comparisons were corrected by controlling FDR. The summary with all statistical analyses are provided in Supplementary Material Table 1.

To determine whether the downstream signaling of PTEN was activated differently according to sex, we analyzed the phosphorylation of AKT, mTOR and FoxO3a. We analyzed the phosphorylation of the AKT threonine 308 residue, which PTEN was previously demonstrated to alter^16^. The quantity of p-AKT^T308^ between the sexes of the wild-type animals did not show any changes (*P* = 0.4553) (Fig. 2a,d), suggesting the existence of a balancing mechanism that leads to similar AKT levels in the two sexes. Curiously, even though no difference was observed in AKT activity, mTOR and FoxO3a proteins demonstrated different basal activity levels depending on sex. Female mice expressed higher levels of the phosphorylated mTOR protein (active form) than did the male mice (*P* < 0.01) (Fig. 2b,d), indicating higher mTor activity in females. In addition, female mice expressed higher levels of the phosphorylated FoxO3a protein (inactive form) than did the male mice (*P* < 0.01), indicating that less FoxO3a resides in the nucleus to regulate gene expression in females than in males (Fig. 2c,d). Thus, these data demonstrated that, under physiological conditions, the activity levels of mTOR and FoxO3a are inversely regulated between the sexes, which occurs independently of AKT activity.

### Autophagy and apoptosis processes are similar in female and male mice under physiological conditions

**Figure 3:**
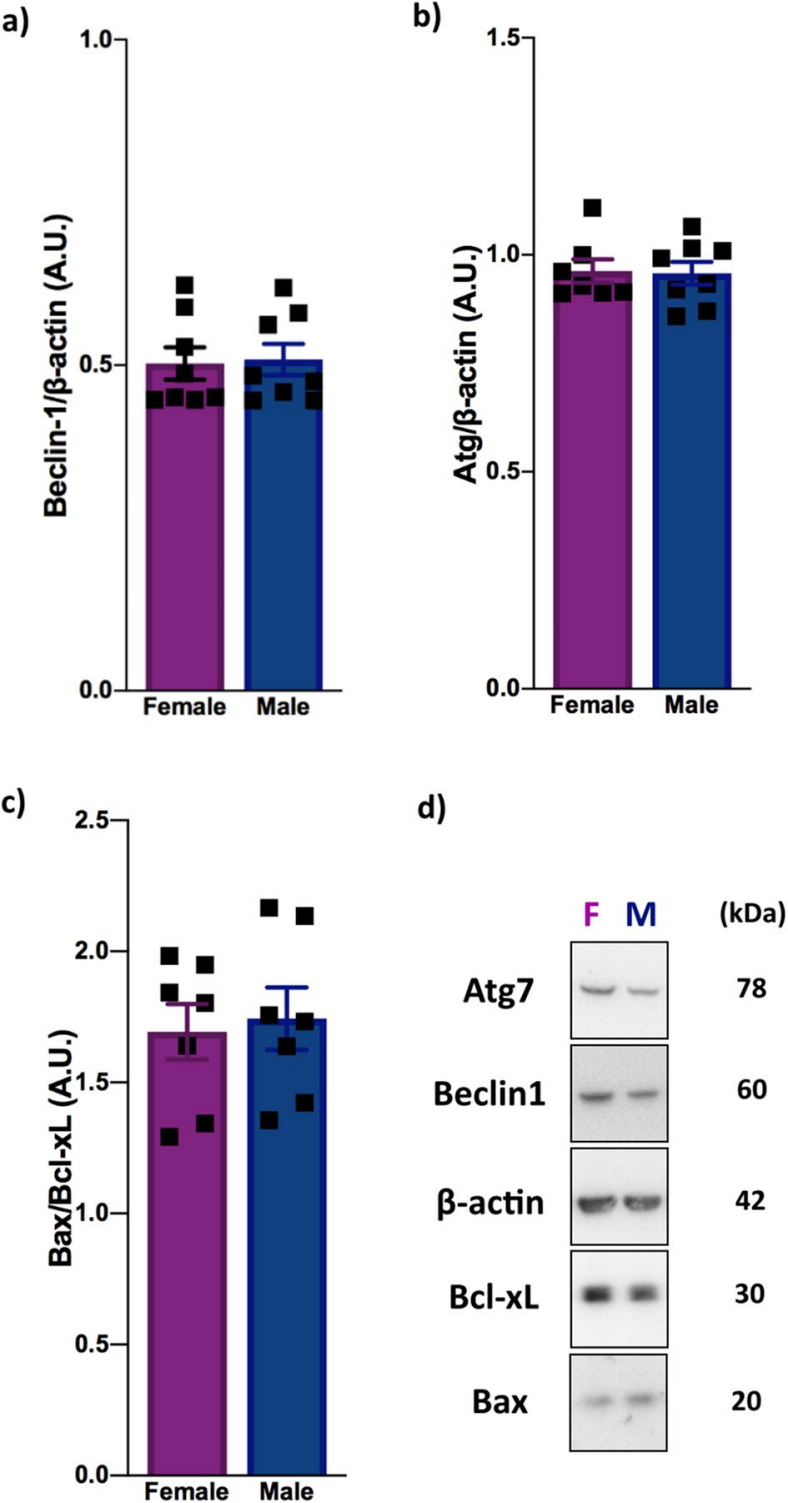
Female and male mice have similar autophagic and apoptotic protein expression profiles. (**a**) Beclin1 protein expression [n = 8 (male and female); ns]. (**b**) Atg7 expression [n = 8 (male and female); ns]. (**c**) The expression ratio of Bax to Bcl-xL [n = 7 (male and female); ns]. (**d**) Representative blot images showing Atg7, Beclin1, Bcl-xL and Bax expression in the male and female mice. All proteins were analyzed in 2-month-old female and male wild-type animals by western blotting. The data were analyzed by two-tailed Student’s *t*-test. All results are expressed as the means ± S.E.M. Full-length blots are provided in Supplementary Fig. S3. Multiple *t* tests comparisons were corrected by controlling FDR. The summary with all statistical analyses are provided in Supplementary Material Table 1.

Due to the importance of autophagy and apoptosis in neurological diseases, as well as differences in mTOR and FoxO3a activity levels in the sexes, we decided to analyze the expression profile of key autophagic and apoptotic proteins. Therefore, we quantified Beclin1, Atg7, Bax and Bcl-xL protein expression in both male and female adult mouse cortices. Interestingly, both sexes expressed similar levels of these proteins (Beclin1 (*P* = 0.8643), Atg7 (*P* = 0.9034); Bax/Bcl-xL (*P* = 0.7637)) (Fig. 3), indicating similar levels of autophagy and apoptosis under physiological conditions.

### The Klotho and PTEN proteins are inversely expressed in both sexes

**Figure 4:**
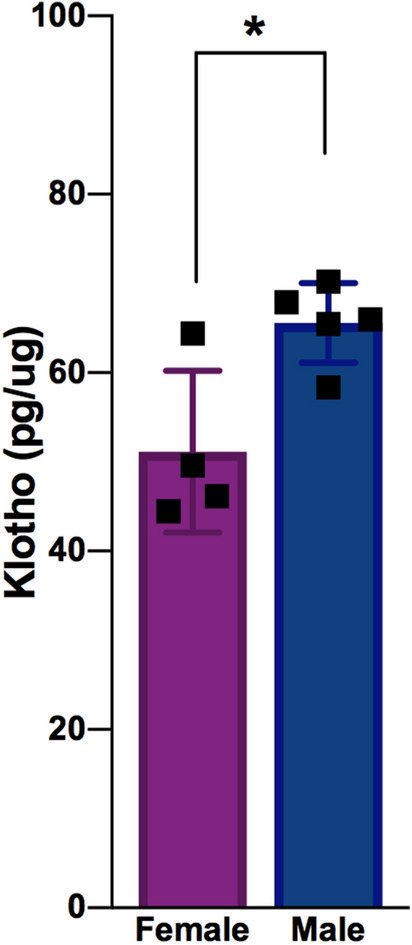
Male mice express more Klotho protein than females. The cortical Klotho protein expression in 2-month-old wild-type animals as analyzed by ELISA [n = 5 (male) and n = 4 (female); *P* = 0.0161, ^#^q = 0.0296]. The data were analyzed by two-tailed Student’s *t*-test, and the results are expressed as the means ± S.E.M. *P < 0.05. Multiple *t* tests comparisons were corrected by controlling FDR. The summary with all statistical analyses are provided in Supplementary Material Table 1.

Since the protein Klotho is known to inhibit insulin signaling and is influenced by mTOR activity, we decided to analyze the levels of Klotho expression in both male and female mice to identify a possible balancing mechanism to explain the similar outcomes. Surprisingly, we found that Klotho protein expression was higher in the male mice than it was in the female mice (*P* < 0.05) (Fig. 4), and that its expression is inversely proportional to that of PTEN in both sexes. Together, these results suggest that Klotho and PTEN may have a complementary functions under physiological conditions and may act together to maintain similar AKT activity levels between the sexes.

## Discussion

Although sex differences^17,18^ and impaired AKT activity^6,19^ are known risk factors for some neurological diseases, very little is known about the influence of sex on this pathway. In this context, our results point to important sex-based differences in key regulators and downstream targets of the PI3K-AKT pathway in the brain under physiological conditions while demonstrating the existence of similar biological processes in male and female systems. These findings may contribute to the understanding of the underlying mechanisms of sex-based responses under triggering situations, perhaps revealing possible cross-talk between PTEN and Klotho expression.

Here, we revealed an important influence of sex on PTEN expression, which was expressed at higher levels in adult female mice than it was in the respective male control (Fig. 1a,c). These data demonstrated that this sex-based influence on PTEN is established later in the adult life of the animal, suggesting that the female brain needs more PTEN than the male brain to maintain its function. Interestingly, another study demonstrated the sex-specific regulation of PTEN in human skeletal muscle, in which women expressed lower PTEN levels than did men^20^. Taken together, these results strengthen the evidence of a sex influence on PTEN regulation, in addition to suggesting that these differences can vary by tissue.

Regarding the PI3K-AKT pathway in the brain, the lack of differences in AKT activity between female and male mice (Fig. 2a) indicates the existence of a balancing regulatory system in both sexes under physiological conditions. Importantly, despite this similar activity levels, our results demonstrated a sex influence on mTOR and FoxO3a phosphorylation, two important downstream targets of the AKT pathway that are highly related to autophagy and apoptosis control, as well as other functions. According to our study, females have higher mTOR activation than males (Fig. 2b), corroborating the finding of another study showing higher mTORC1 activation in the liver and heart tissue of females^21^. Together, these results suggest that this sex-based activity occurs in an organ-independent manner and supports the idea that female systems require higher mTOR activity. Moreover, our findings showing that females have higher basal mTOR activity than males may contribute to understanding some of the risk factors for diseases such as AD, which is known to have a higher incidence in women and has been linked with increased mTOR activity and decreased autophagy^22^. On the other hand, our results showed lower FoxO3a activity in female mice than in male mice (Fig. 2c), suggesting an opposite physiological need for mTOR and FoxO3a function between the sexes. Although, to the best of our knowledge, no data show an influence of sex on FoxO3a activity, the deregulation of FoxO3a activity is related to AD^23^ and PD^24^, and this sex-influenced activity may help to explain the different incidence rate according to sex. Moreover, our results demonstrated that, despite the different basal activity levels of mTOR and FoxO3a, proteins such as Beclin1, Atg7, Bax and Bcl-xL, which play key roles in autophagy and apoptosis, are similarly expressed in both sexes under physiological conditions (Fig. 3), suggesting the existence of another sex-based pathway with a mechanism that balances the outcomes of these processes. However, it is likely that, under AKT hyperactivation conditions, for example, each sex responds differently due to their distinct basal mTOR and FoxO3a activity levels, resulting in a sex-dependent autophagic and/or apoptotic response.

Our results showed a higher level of Klotho expression in the male than in the female mice (Fig. 4), leading to a surprisingly inverse expression profile of Klotho and PTEN between the sexes. Since Klotho is also known to inhibit insulin signaling and the AKT pathway, these data may help to explain the interesting lack of difference in AKT activity between female and male mice, suggesting that these proteins may work together to maintain a physiological balance in both systems. In fact, a previous study indicated that women express lower levels of Klotho in the cerebrospinal fluid than do men, in agreement with our findings^25^. In addition, higher Klotho expression seems to be related to lower mTOR activity^26^, which may be the reason for the increased expression of Klotho observed in the male mice than in the female mice. These findings make it reasonable to hypothesize that the different mTOR activity in the sexes plays a role in counterbalancing the sex differences in PTEN expression and contributing to AKT balance by regulating Klotho expression. Moreover, it is also possible that some sex-based responses under stress situations are due to this difference in Klotho expression.

**Figure 5:**
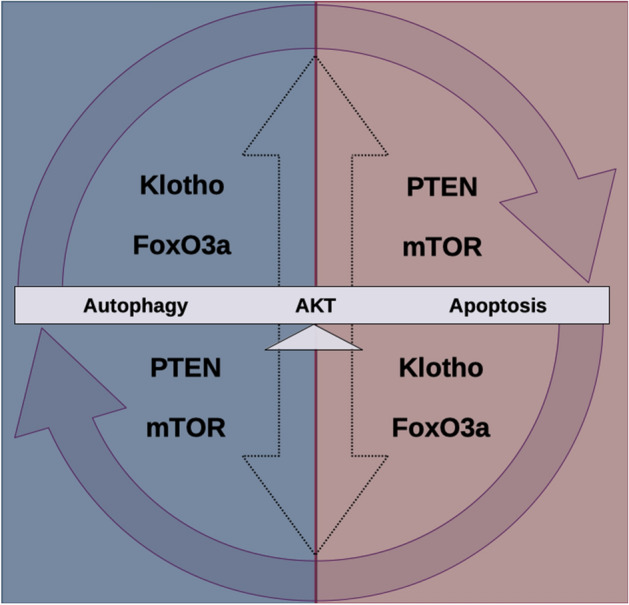
Proposed sex differences influence the maintenance of physiological conditions. Female and male physiological systems show inverse expression of PTEN and Klotho protein and different levels of mTOR and FoxO3a activity. However, comparisons of both sexes show no differences in AKT activity or apoptosis and autophagy processes, suggesting that these sex differences may be contributing to balancing mechanisms that establish similar outcomes between them.

In conclusion, our data demonstrated important sex-based differences in PTEN and Klotho expression and in mTOR and FoxO3a activity, which may lead to a balance in AKT activity with autophagy and apoptosis in the sexes under physiological conditions (Fig. 5). Despite the limitation of the small sample size, these data can bring new and important perspectives of the possible relationship between PTEN and Klotho in male and female mice, which will likely open new questions for further investigation. These findings can have important contributions for understanding a possible mechanism underlying sex-based responses and the incidence of neurological diseases, as well as in the search for new and more specific target treatments. Considering the lack of studies comparing the maintenance of these pathways in the sexes, especially in the CNS, we highlight the need of more and larger studies to confirm and help to elucidate the possible complementary functions and expression of PTEN and Klotho, making them targets in which the absence of one can be counterbalanced by hyperexpressing the other. Moreover, since several studies have pointed to impairments in the cell death processes in neurodegenerative diseases and that intervention in this mechanistic pathway seems to be a promising therapeutic strategy, further investigation of the differences in mTOR and FoxO3a activity between the sexes may also contribute to a sex-specific target for drug development.

## Methods

### Mice

The *Pten*^loxP/+^;*Nse*-*Cre*^+^ lineage mice generated by crossing *Pten*^loxP/loxP^ mice (donated by Dr. Antonio Di Cristofano from Albert Einstein College of Medicine, Bronx, NY, USA) with *Nse-Cre*^+^ mice (B6.Cg-Tg(Eno2-cre)39Jme/J mice, Jackson Laboratory, Bay Harbor, ME, USA), with a C57Bl/6J background. All male and female wild-type mice (*Pten*^+/+^; *Nse*-*Cre*^+^, *Pten*^loxP/loxP^; *Nse*-*Cre*^-^ and *Pten*^loxP/+^; *Nse*-*Cre*^-^) used in the experiments were generated by crossing *Pten*^loxP/+^; *Nse*-*Cre*^+^ and *Pten*^loxP/+^;*Nse*-*Cre*^-^ mice. Male and female animals were maintained in plastic microisolation cages in groups of as many as 5 animals at 22 ± 2 °C in a 12-h/12-h light/dark cycle at the same animal facility of the Laboratory of Molecular Neuropharmacology (Department of Pharmacology, Institute of Biomedical Sciences, University of São Paulo, São Paulo, Brazil) with food and water available ad libitum. Euthanasia before dissection of the cortex of 2-month-old male and female mice was performed on the same day or at the same time on different days. In addition, to reduce variability among the female mice, using mice from the same cage was a priority. All experimental procedures were approved by and performed under the regulation of the Ethical Committee for Animal Research of the Institute of Biomedical Sciences (CEUA/ICB/USP, protocol n° 76/2016) and were in accordance with the guidelines of the Brazilian Society of Laboratory Animals Science (SBCAL).

### Tissue protein extraction

Cortical tissues were homogenized in a glass-glass Dounce homogenizer in ice-cold lysis buffer (20 mM HEPES, 1.0 mM MgCl_2_, 0.5 mM EDTA, 1% NP-40, 1.0 mM EGTA, 0.5 mM PMSF, 2 g/mL leupeptin, 2 g/mL antipain, 3 mM Na_3_VO_4_, and 20 mM sodium pyrophosphate) and centrifuged at 17,000×*g* for 5 min at 4 °C. The supernatant was collected and stored at − 80 °C for western blot analysis. The protein concentration was determined using the Bradford colorimetric method (#500-0006, Bio-Rad, Hercules, CA, USA).

### Western blotting

Protein extracts were adjusted to a final concentration of 2.0 µg/µL in sample buffer (125 mM Tris–HCl, 4% SDS, 20% glycerol, 200 mM DTT, and 0.02% bromophenol blue at pH 6.8) and subjected to SDS-PAGE electrophoresis using 10% polyacrylamide gel. The proteins were transferred onto nitrocellulose membranes, blocked with 5% BSA, and incubated with primary antibodies against the following targets: PTEN (Cell Signaling Technology, 1:1000), AKT (Santa Cruz Biotechnology, 1:2000), p-AKT^T308^ (BD Biosciences, 1:750), mTOR (Cell Signaling, 1:1000), p-mTOR (Cell Signaling, 1:1000), FoxO3a (Cell Signaling, 1:1000), p-FoxO3a (Cell Signaling, 1:1000), Beclin1 (Cell Signaling, 1:500), Bcl-xL (Cell Signaling, 1:333), Bax (Cell Signaling, 1:333), Atg7 (Cell Signaling, 1:500), and β-actin (Sigma-Aldrich, 1:10,000). Primary antibodies were diluted in 1% BSA, while secondary antibodies were diluted in 5% BSA (1:2000).

### Embryonic neuronal cell culture

Primary neuronal cultures were prepared from cerebral cortices of embryonic day (E) 16.5 from male and female WT embryos. Briefly, after the meninges were removed, cortices were digested with 0.05% trypsin for 20 min at 37 °C. the Cells were dissociated with a glass pipette in the presence of DNase (Sigma-Aldrich, #9003-98-9). The dissociated cells were plated at a density of 1.0 × 10^5^ cells/mL on polyethyleneimine (PEI, Sigma-Aldrich, #9002-98-6)-coated wells and cultured in MEM (VITROCELL) containing 10% FBS for 3 h. After seeding, the medium was changed to neurobasal medium (Thermo Fisher, #21103049) supplemented with B-27 (Thermo Fisher, #17504044), 0.5 mM glutamine and 1% penicillin/streptomycin. The cells were incubated at 37 °C in a humidified chamber with 95% air and 5% CO_2_ and used for experiments after 7 or 14 days in vitro (DIV).

### Polymerase chain reaction

Embryo tails were collected immediately after dissection and processed to identify their sex. DNA was extracted in 75 µL of 25 mM NaOH and 0.2 mM EDTA for 1 h at 98 °C on an orbital shaker (900 rpm). An equal volume of 40 mM Tris HCl, pH 5.5, was added, and the solution was centrifuged for 3 min at 1500×*g*. The supernatant was collected and stored at − 80 °C. The PCR protocol for embryo sex determination was adapted from McFarlane et al.^27^, using the primer pairs SX_F: 5′-GATGATTTGAGTGGAAATGTGAGGTA-3′ and SX_R: 5′-CTTATGTTTATAGGCATGCACCATGTA-3′. Briefly, the reactions were performed in a final volume of 20 µL solution of 10 mM Tris–HCl pH 8.3, 50 mM KCl, 1.5 mM MgCl_2_, 0.2 mM dNTPs, 0.2 µM primers, and 1 unit Taq polymerase (New England Biolabs, Mass, USA) with the following parameters: initial denaturation at 95 °C for 3 min, 35 cycles of 94 °C for 15 s, 55 °C for 30 s, and 72 °C for 40 s, followed by final elongation at 72 °C for 5 min.

### Immunofluorescence

After 7 or 14 days in vitro, the medium was removed from the plate, and the coverslips were washed twice with cold PBS. The cells were fixed in 4% formaldehyde for 15 min at 4 °C and incubated in blocking solution (5% donkey serum; 0.01% Triton X-100 in PBS) for 1 h at 25 °C prior to overnight incubation with rabbit anti-PTEN antibody (1:200, Cell Signaling Technology, #138G6) at 4 °C. The cells were washed with PBS three times for 10 min each time and then incubated with anti-rabbit Alexa Fluor 488 (1:1000, Life Technology, #A21206) for 2 h at 25 °C. DAPI (1:25,000) was used to stain nuclei. Images were captured under a fluorescence microscope (Nikon 80i, Imaging Software–NIS Element V4.6) using a 20× objective and analyzed with ImageJ (NIH) software.

### Immunofluorescence measurement

Immunofluorescence was measured using ImageJ, as described previously^16^. In brief, the total corrected cellular fluorescence (TCCF) was determined as TCCF = integrated density – (area of selected cell × mean fluorescence of background readings). TCCF was calculated for approximately 50 cells in each embryo. The average TCCF of all the obtained values is reported.

### ELISA kit

The klotho protein concentration in the cortices from the 2-month-old mice was assessed by using a mouse KL ELISA kit (#OKEH01597, Aviva Systems Biology, CA, USA). Equal amounts of protein were utilized for the Klotho ELISA following the manufacturer’s specifications.

### Statistical analysis

The results are expressed as the means and standard deviation in graph bars in the form of dot plots. Parametric analyses were conducted through two-tailed Student’s *t*-test or two-way ANOVA followed by Tukey’s post hoc test. Multiple *t* tests were corrected by controlling the False Discovery Rate (FDR) using the Two-stage step-up method of Benjamini, Krieger and Yekutieli with desired FDR (Q) = 5%. All statistical analyses were performed using GraphPad Prism version 6.01 for Windows (GraphPad Software, La Jolla, CA, USA).

## Supplementary information

Supplementary Information.

## Data Availability

All data generated during this study are included in this article and its Supplementary Information file.
