# Supplementary material for: Inverse sex-based expression profiles of PTEN and Klotho in mice
Source: Sci Rep. 2020 Nov 19;10:20189. doi: 10.1038/s41598-020-77217-5 (PMC7677532; doi:10.1038/s41598-020-77217-5)
Supplement: Supplementary file 1 — Supplementary Information. [file 41598_2020_77217_MOESM1_ESM.pdf]

Inverse sex-based expression profiles of PTEN and Klotho in mice

Natalia Prudente de Mello[1], Diana Zukas Andreotti[1,2], Ana Maria Orellana[2], Cristoforo Scavone[2], Elisa Mitiko Kawamoto[1]

[1]Laboratory of Molecular and Functional Neurobiology, Department of Pharmacology, Institute of Biomedical Sciences, University of São Paulo, Brazil.
[2]Laboratory of Molecular Neuropharmacology, Department of Pharmacology, Institute of Biomedical Sciences, University of São Paulo, Brazil.
Correspondence and requests for materials should be addressed to E.M.K (email: kawamotoe@gmail.com)

SUPPLEMENTARY MATERIAL

Supplementary Figures

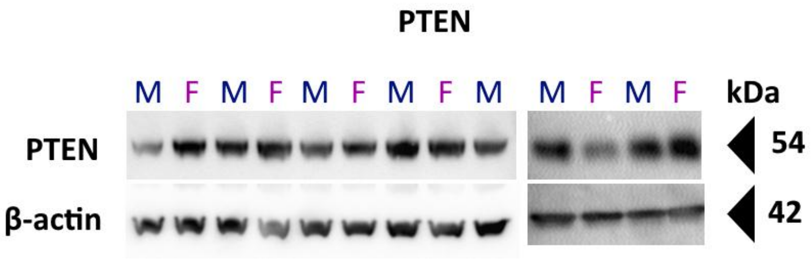

Supplementary Figure S1. Western blotting membrane images of cortical PTEN protein [n = 7 (male) and n = 6 (female)]. Approximate position of the molecular weight standard is indicated by the black arrowhead. Full membrane blots are provided in Supplementary Information Figure 1.

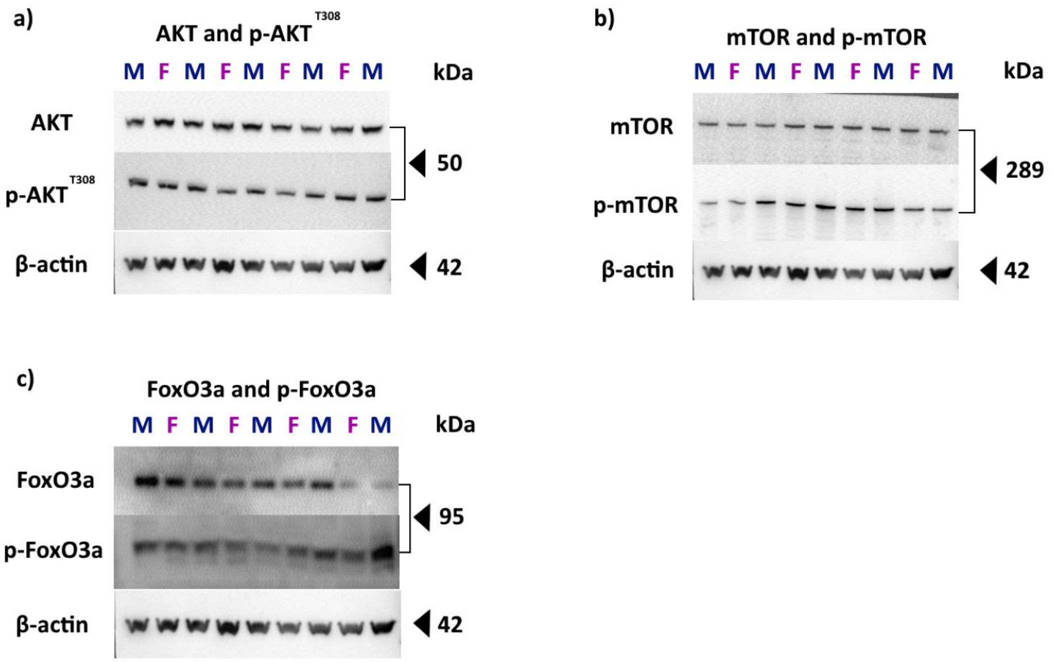

Supplementary Figure S2. Western blotting membrane images of AKT, mTOR and FoxO3a. **(a)** AKT and p-AKT^{T308} [n = 5 (male) and n = 4 (female); ns]; **(b)** mTOR and p-mTOR [n = 5 (male) and n = 4 (female); *P* = 0.0011]; **(c)** FoxO3a and p-FoxO3a [n = 5 (male) and n = 4 (female); *P* = 0.0020]. Approximate position of the molecular weight standard is indicated by the black arrowhead. Full membrane blots are provided in Supplementary Information Figure 2.

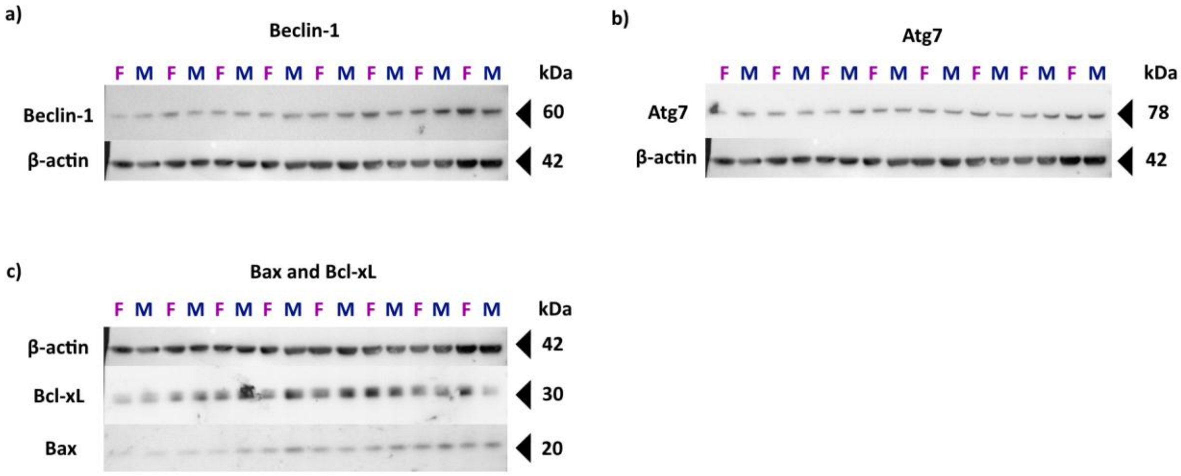

Supplementary Figure S3. Western blotting membrane images of Beclin-1, Atg7, Bax and Bcl-xL. **(a)** Beclin-1[n = 8 (male and female); ns]; **(b)** Atg7 [n = 8 (male and female); ns]; **(c)** Bax and Bcl-xL [n = 7 (male and female); ns]. Approximate position of the molecular weight standard is indicated by the black arrowhead. Full membrane blots are provided in Supplementary Information Figure 3.

Supplementary Table

Protein	Statistical Analysis	Group compared	F (DFn, DFd)	P Value	Multiple Comparisons correction	Adjusted P Value or q Value	Significant or Discovery
**PTEN**	Multiple t test	F x M	-	0.033520	FDR	0.041062	Yes
	Two-way ANOVA	Interaction	F(1, 11) = 18.13	0.0013	-	-	Yes
		DIV7 x DIV14 (Time Point)	F(1, 11) = 43.73	<0.0001	-	-	Yes
		F x M (Sex)	F(1, 11) = 11.71	0.0057	-	-	Yes
		DIV7: F x M	-	-	Tukey	0.9251	No
		DIV14: F x M	-	-	Tukey	0.0014	Yes
		F: DIV7 x DIV14	-	-	Tukey	<0.0001	Yes
		M: DIV7 x DIV14	-	-	Tukey	0.4148	No
**AKT**	Multiple t tests	F x M	-	0.463137	FDR	0.425507	No
**p-AKT^{Thr308}/AKT**	Multiple t tests	F x M	-	0.455328	FDR	0.425507	No
**mTOR**	Multiple t tests	F x M	-	0.026126	FDR	0.038406	Yes
**p-mTOR/mTOR**	Multiple t tests	F x M	-	0.001147	FDR	0.007303	Yes
**FoxO3a**	Multiple t tests	F x M	-	0.006478	FDR	0.015871	Yes
**p-FoxO3a/FoxO3a**	Multiple t tests	F x M	-	0.001987	FDR	0.007303	Yes
**Beclin1**	Multiple t tests	F x M	-	0.864294	FDR	0.603663	No
**Atg7**	Multiple t tests	F x M	-	0.903441	FDR	0.603663	No
**Bax/Bcl-xL**	Multiple t tests	F x M	-	0.763670	FDR	0.603663	No
**Klotho**	Multiple t tests	F x M	-	0.016106	FDR	0.029595	Yes

Supplementary Table 1. Summary of the statistical analyses and respective values. Analyses were conducted through two-tailed Student's *t*-test corrected by False Discovery Rate (FDR), or two-way ANOVA followed by Tukey's post hoc test. All adjusted P values (Tukey) or q values (FDR) and the respective significance or discovery statuses are included in the table.

Inversed sex-based expression profiles of PTEN and Klotho in mice

Natalia Prudente de Mello[1], Diana Zukas Andreotti[1,2], Ana Maria Orellana[2], Cristoforo Scavone[2], Elisa Mitiko Kawamoto[1]

Supplementary Information

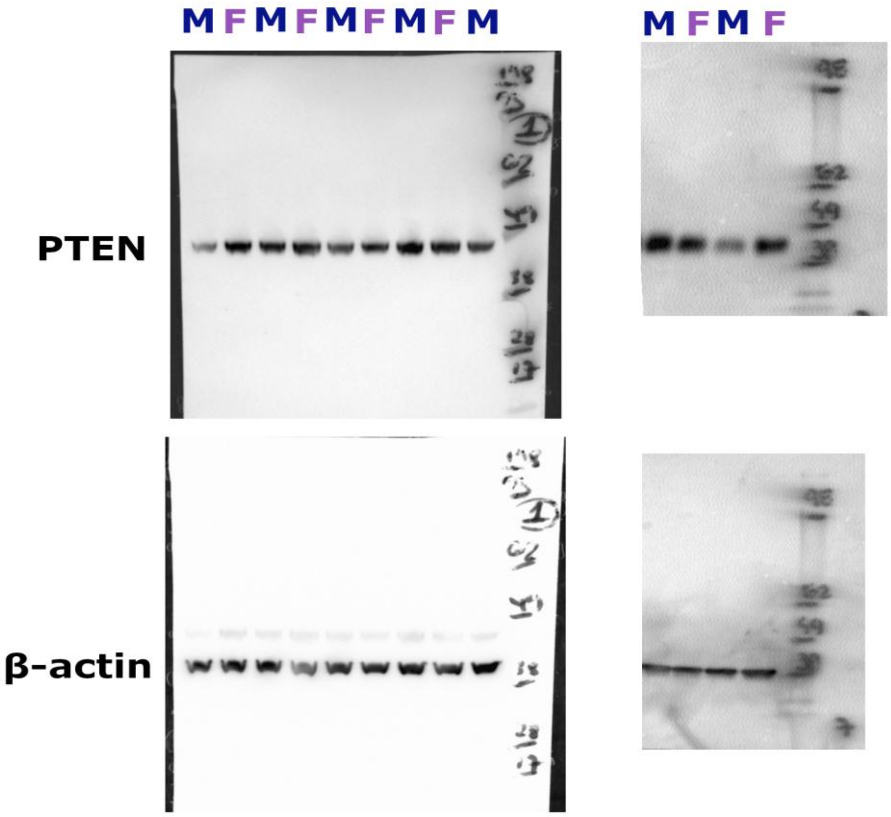

Supplementary Information - Figure 1

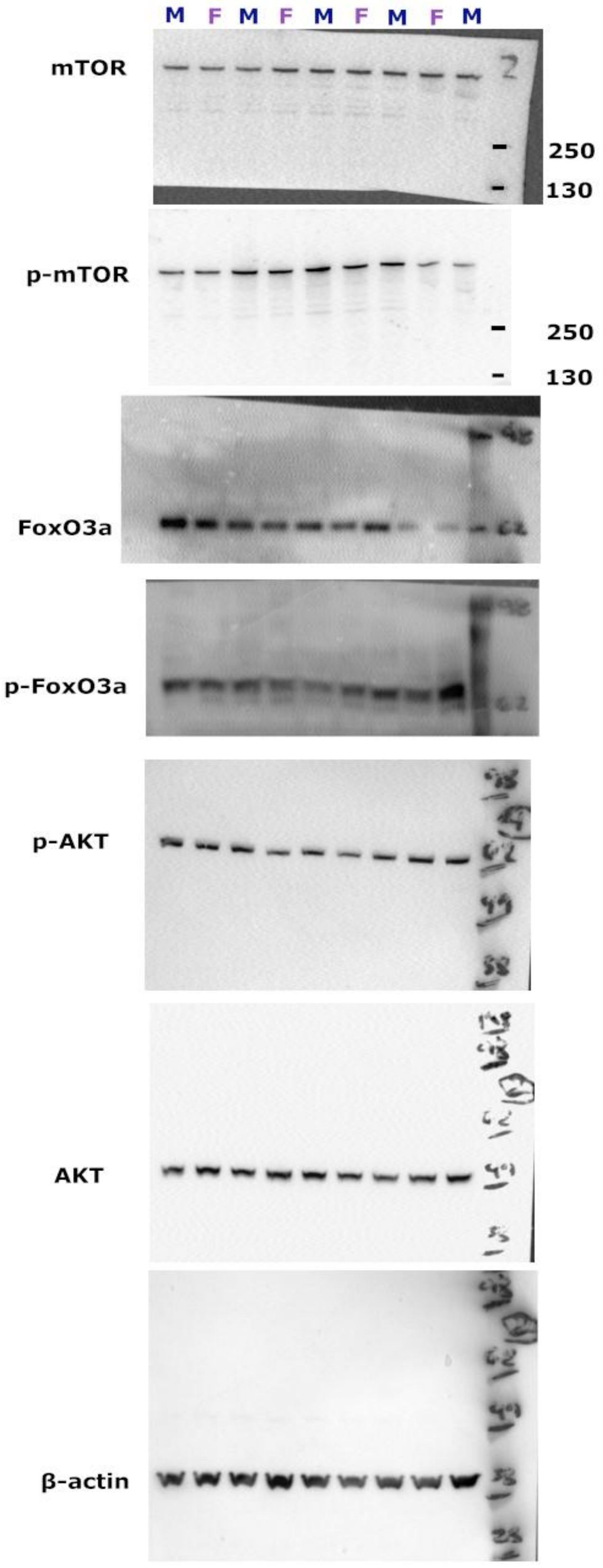

Supplementary Information - Figure 2

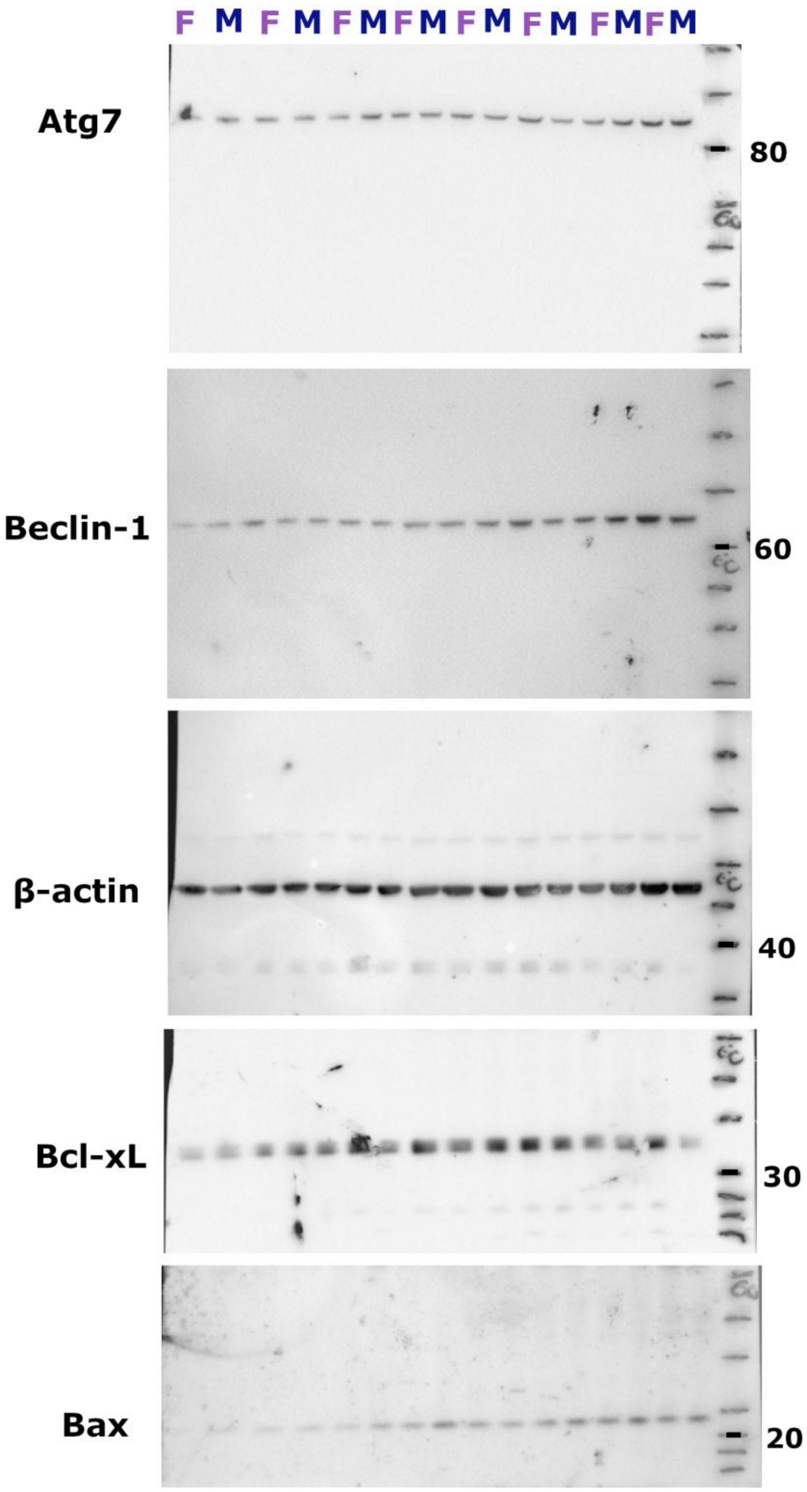

Supplementary Information - Figure 3
